# Evaluation of corneal elastic modulus based on Corneal Visualization Scheimpflug Technology

**DOI:** 10.1186/s12938-019-0662-1

**Published:** 2019-04-04

**Authors:** Xiao Qin, Lei Tian, Haixia Zhang, Xinyan Chen, Lin Li

**Affiliations:** 1 Beijing Key Laboratory of Fundamental Research on Biomechanics in Clinical Application, Capital Medical University, Beijing, 100069 China; 2 School of Biomedical Engineering, Capital Medical University, Beijing, 100069 China; 3 Beijing Ophthalmology & Visual Sciences Key Laboratory, Beijing Institute of Ophthalmology, Beijing Tongren Eye Center, Beijing Tongren Hospital, Capital Medical University, Beijing, China

**Keywords:** Corneal Visualization Scheimpflug Technology (Corvis ST), Reisner’s theory, Cornea, Elastic modulus, Intraocular pressure (IOP)

## Abstract

**Background:**

Corneal biomechanical properties are important for the diagnosis of corneal diseases, individualized design and prognosis of corneal surgery. Clinical available devices such as Ocular Response Analyzer (ORA) and Corneal Visualization Scheimpflug Technology (Corvis ST) can provide corneal biomechanics related parameters, while corneal elastic modulus cannot be extracted directly from them at present. The aim of this study is to suggest a method to determine corneal elastic modulus based on the results of Corvis ST test according to Reissner’s theory on the relation between stress and small displacement in shallow spherical shell.

**Results:**

Five rabbits (10 eyes) and 10 healthy humans (20 eyes) were measured with Corvis ST to obtain the normal range of corneal elastic modulus. Results showed Corneal elastic modulus of rabbit was 0.16 MPa to 0.35 MPa, human corneal elastic modulus was 0.16–0.30 MPa. Rabbit corneas were also measured at different intraocular pressures (IOP), and results showed corneal elastic modulus, first applanation time (A1T) and stiffness parameter (SP-A1) were positively correlated with IOP. Deformation amplitude (DA), the second applanations time (A2T), and peak distance (PD) were negatively correlated with IOP. Finite element method was used to simulate the Corvis measurements according to the calculated elastic modulus and the simulated corneal apical displacements were agreement with experimental results in general.

**Conclusions:**

The method to determine corneal elastic modulus based on Corvis test according to the relationship between force and displacements of shallow spherical shell is convenient and effective.

## Background

Cornea is the soft tissue located in the outer layer of the eyeball. The transparent cornea provides 70% ocular refractive power [1]. Corneal biomechanical properties play an important role in maintaining the normal shape and function of cornea. Besides, the occurrence and development of ocular diseases such as keratoconus, myopia and pseudoexfoliation syndrome, the design and prognosis of corneal surgeries such as corneal transplantations, corneal refractive surgery and corneal collagen crosslinking surgery are also correlated to corneal biomechanics [1–7]. Therefore, more and more ophthalmologists and researchers expect to obtain human corneal biomechanics though simple clinical measurements.

Similar to most biological tissues, corneal biomechanics include its anisotropic, nonlinear elastic properties and viscoelastic properties. Corneal nonlinear elastic properties often be described as corneal tangent modulus under certain stress. While studies have found that cornea can be regarded as linear elastic material under physiological state [7], so the mechanical properties of cornea under physiological state may be described by elastic modulus, or Young’s modulus, as most of researchers concerned.

Ocular Response Analyzer (ORA) and Corneal Visualization Scheimpflug Technology (Corvis ST) are two of the most widely used devices for measurements of corneal biomechanical properties in clinic. In fact, the parameters provided by these devices such as corneal hysteresis (CH) and corneal resistance factor (CRF) by ORA measurements and corneal stiffness parameter (SP-A1), corneal deformation amplitude (DA) and first applanation time (A1T), etc. by Corvis measurements are descriptions of mechanical process of cornea under air-puff. These parameters are related to corneal biomechanics, intraocular pressure (IOP) and corneal geometrical parameters [8–10], so we call them corneal biomechanics related parameters. Although many researchers attempted to get the relationship between these parameters and mechanical properties such as Young’s modulus, there is still lack of acknowledged results at present [11, 12].

Many studies [2, 13–25] have put great concerns on how to extract corneal elastic modulus directly from Corvis and/or ORA parameters. Glass [13] and Han [14] regarded cornea as a simplified spring and dashpot system to simulate corneal response to air-puff. These studies can help researchers to understand corneal biomechanical related parameters preliminary, but cannot determine corneal biomechanical properties quantitatively due to too simple models. Besides, researches have simulated corneal response under air-puff pressure by finite element method [16–22] in order to evaluate corneal biomechanical properties based on different corneal constitutive model such as Fung’s model [18], Ogden model [19], fiber-dependent model [20] or other models [21, 22]. These studies provided approaches to obtain corneal traditional biomechanical properties based on Corvis results while these methods have a higher requirement for corneal geometrical models and are time consuming, which make it difficult to be used in clinic directly. Furthermore, Wang et al. [2] detected air puff-time curve and corneal apical displacement–time curve, from which two parameters: corneal tangent stiffness coefficient (*S*_TSC_) and corneal energy absorbed area (*A*_absorbed_) were determined. However, both of *S*_TSC_ and *A*_absorbed_ are not corneal elastic modulus or viscoelastic parameter. In the latest version of software of Corvis, Stiffness Parameter **(**SP-A1) is provided which is expressed by the ratio of the force and displacement at the first applanation state [23]. From the expression of SP-A1 we can find the SP-A1 was affected by IOP. These two parameters are significantly correlated with corneal geometrical parameters which lead to the difficulty to evaluate corneal elastic property accurately. In addition, Shih et al. [24] estimated corneal Young’s modulus in vivo by proposing modified Taber’s model to describe the relationship between the force applied on cornea and corneal apical displacement based on Taber’s results [25]. While in their paper cornea was regarded as hemisphere and the air-puff pressure was assumed as a point load at the apical point of cornea, which have discrepancies with corneal normal geometrical shape. Therefore, corneal biomechanical properties such as its elastic and viscoelastic properties have not been directly determined from Corvis parameters yet.

Considering relatively small deformation of the cornea and small range of the IOP, the cornea can be regarded as linear elastic material during Corvis measurements [7]. Motivated by the reported works [26, 27] on ultrasound indentation techniques, we applied Reissner’s theory [28] which described the stresses and small displacements in shallow spherical shells to determine corneal elastic modulus. The present study applied Reissner’s theory [28] to calculate corneal elastic modulus from Corvis measurements on human and rabbits in vivo. Besides, rabbit eye balls were also measured in vitro under different IOPs to evaluate the IOP’s influence on the calculated corneal elastic modulus. To validate the accuracy of the calculated elastic modulus, Corvis test was simulated using explicit finite element method based on the calculated corneal elastic modulus and corneal apical displacements were extracted to be compared with the experimental data. The significance of this work is to provide an effective method to determine corneal elastic modulus from the output data of Corvis test in clinic directly and may give possible guidance for the diagnosis of corneal disease and design of corneal surgery.

## Materials and methods

### Method to evaluate corneal modulus

**Fig. 1:**
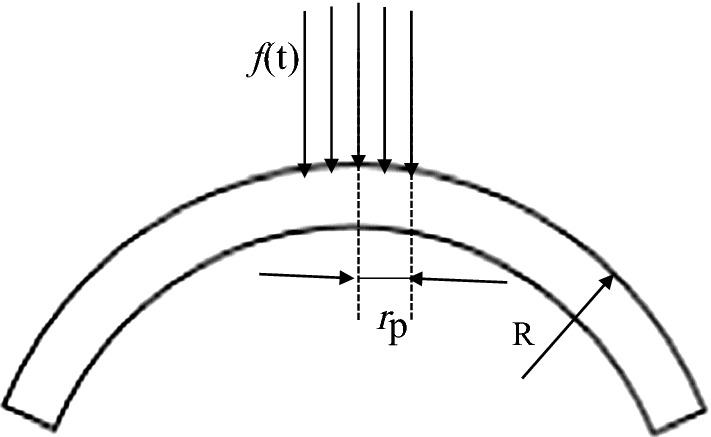
Simplified corneal geometrical model and the air-puff force located on it

As Corvis test can provide the corneal response under rapid air-puff on corneal apex, we can regard it as indentation experiments and corneal elastic modulus can be determined by the air-puff forces-corneal apical displacements curve. The cornea was taken as a shallow spherical shell as showed in Fig. 1 in this study, and the air-puff from Corvis was regarded as a surface load act on corneal apex whose area is a circle with radius of *r*_p_. As *r*_p_ is small enough compared to the whole cornea, we considered there was no edge restraint at limbus. We used the reported air-puff force (Eq. 1) [2] instead of air-puff force at the gas nozzle as the energy loss from Corvis gas nozzle to corneal surface; *r*_p_ was replaced by the radius of first applanation area; *R* is corneal radius which was extracted by detecting corneal anterior surface edge with threshold segmentation based on Canny operator and circular fitting of the edge. Besides, corneal apical displacements were also detected based on the results of corneal anterior edge detection.

1 $$f(t) = 36.04e^{{ - \left( {\frac{t - 15.54}{5.119}} \right)^{2} }} + 9.799e^{{ - \left( {\frac{t - 9.642}{5.119}} \right)^{2} }}$$

When the air-puff acted on the cornea, cornea deformed and the relationship between corneal apical displacements and the concentrated force *f* is as follows [28]:

2 $$\delta = - \frac{{f(R - t/2)\sqrt {12(1 - \nu^{2} )} }}{{\pi {\kern 1pt} E{\kern 1pt} t^{ 2} }}\frac{{c_{1} + c_{5} }}{{\mu^{2} }}$$

In which $\nu$ represents corneal Poisson’s ratio and was set to 0.49 in this study; *t* is the central corneal thickness; $E$ is corneal elastic modulus and $\mu$ is defined as Eq. 3:

3 $$\mu = r_{p} \left[\frac{{12(1 - \nu^{2} )}}{{(R - t/2)^{2} t^{2} }}\right]^{1/4}$$

According to Reissner’s results, *c*_1_ and *c*_5_ were determined by the following equations from Eq. 4a to Eq. 4e:

4a $$c_{{1{\kern 1pt} }} {\text{ber }}\mu + c_{{2{\kern 1pt} }} {\text{bei }}\mu - c_{{3{\kern 1pt} }} \ker \mu - c_{{4{\kern 1pt} }} {\text{kei }}\mu = - c_{5}$$

4b $$c_{1} {\text{ber}}^{\prime } \, \mu + c_{2} {\text{bei}}^{\prime } \, \mu - c_{3} {\text{ker}}^{\prime } \, \mu - c_{4} {\text{kei}}^{\prime } \, \mu = 0$$

4b $$- c_{{1{\kern 1pt} }} {\text{bei}}\, \mu + c_{{2{\kern 1pt} }} {\text{ber}}\, \mu + c_{{3{\kern 1pt} }} {\text{kei}}\, \mu - c_{{4{\kern 1pt} }} { \ker }\, \mu = 0$$

4d $$c_{{1{\kern 1pt} }} {\text{bei}}^{\prime } \, \mu - c_{{2{\kern 1pt} }} {\text{ber}}^{\prime } \, \mu - c_{{3{\kern 1pt} }} {\text{kei }}\mu + c_{{4{\kern 1pt} }} { \ker }^{\prime } \mu = 0$$

4e $$c_{1} {\text{ber}}\,\mu + c_{2} {\text{bei}}\,\mu - c_{3} \ker \,\mu - c_{4} {\text{kei}}\,\mu = 1$$

In these equations, ber, bei, ker, kei are Kelvin functions. It is obvious that *c*_*5*_= − 1. And then we can calculate corneal elastic modulus by the Eq. 5:

5 $$E = \frac{{f(R - t/2)\sqrt {12(1 - \nu^{2} )} }}{{\pi {\kern 1pt} \delta {\kern 1pt} t^{ 2} }}\frac{{1 - c_{1} }}{{\mu^{2} }}$$

**Fig. 2:**
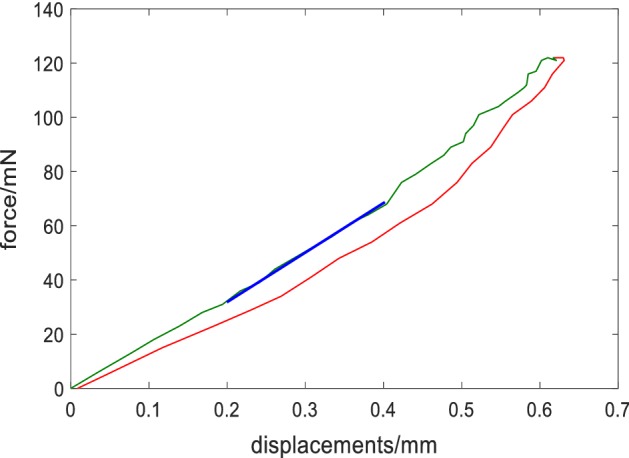
Procedure to determine corneal elastic modulus based on Corvis test

The whole procedure to determine corneal elastic modulus was showed as Fig. 2.

**Fig. 3:**
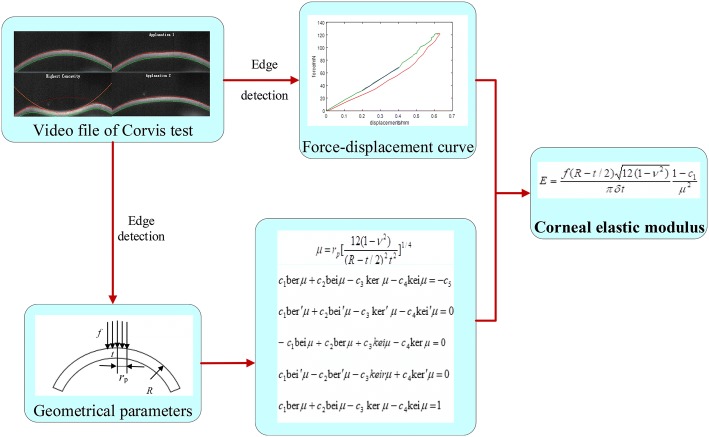
Force-displacement curve of corneal Corvis test. The green and red curve represent the loading and unloading curve, respectively

The corneal apical displacements at the first applanation state were between 0.1 and 0.2 mm, and the air puff forces-corneal apical displacements showed a better linearity when the corneal apical displacements less than 0.5 mm. So, in this study, the air-puff force and corneal apical displacements curve was plotted and the slope when the corneal vertex displacement was between 0.2 to 0.4 mm during loading process (Fig. 3) as the contact radius (*r*_*p*_) between the air-puff and cornea is relative constant.

### Subjects and measurements

**Fig. 4:**
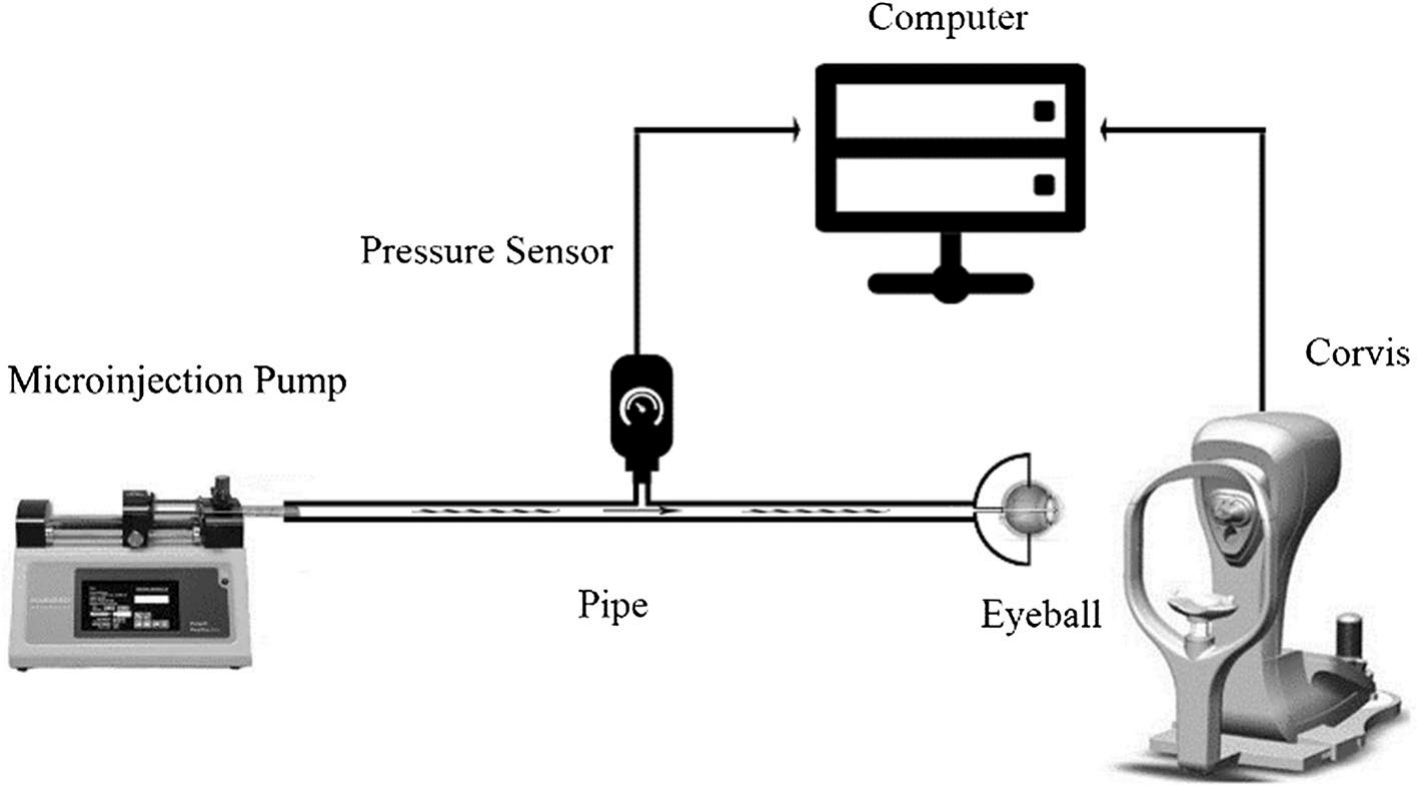
Platform of eyeball inflation and Corvis test

Five normal New Zealand rabbits (10 eyes) and ten healthy subjects (20 eyes) were enrolled in this study. Inclusion criteria for normal rabbits and subjects are as follows: all of the rabbits were provided by Animal Laboratory Center of Capital Medical University, the rabbits aged 7 months, the weight of the rabbits were between 3.5 and 4.0 kg, the intraocular pressures were not higher than 20 mmHg, ocular disease were not found in all rabbits; the age of the subjects were between 20 and 25 years, patients with high myopia, keratoconus or other ocular diseases were excluded, all was the subjects have no history of refractive surgery or other ocular surgeries. The experiments followed with the ARRIVE guidelines and NIH guidelines. All measurements on human eyes were noninvasive and were in accordance with the Code of Ethics of the World Medical Association. All of the experiments were approved by the ethics committee of Capital Medical University. All subjects have signed the consent and were informed consent. All rabbit eyes and human eyes were measured with Corvis ST in vivo. After that we extracted the rabbits’ eyeballs and fixed them on an experimental platform of eyeball inflation as showed in Fig. 4, where we also referred to the reported platform [31]. A vein indwelling needle with radius of 0.5 mm was inserted from optic nerve into the anterior chamber to adjust the intraocular pressure. And the IOP was measured with the Pclab Biomedical signal acquiring processing system (Beijing Microsignalstar technology development CO., LTD). The IOP was controlled by injecting physiological saline into the anterior chamber at a speed of 20 μl/min. Corvis tests were carried out when the IOPs were stable at 7, 14, 21, 28, 35 mmHg. The tests were controlled within 5–8 min and no significant dehydration was found. All Corvis measurements were taken by the same technician and captured by automatic release to ensure the absence of user dependency.

### Finite element method to validate corneal elastic modulus

**Fig. 5:**
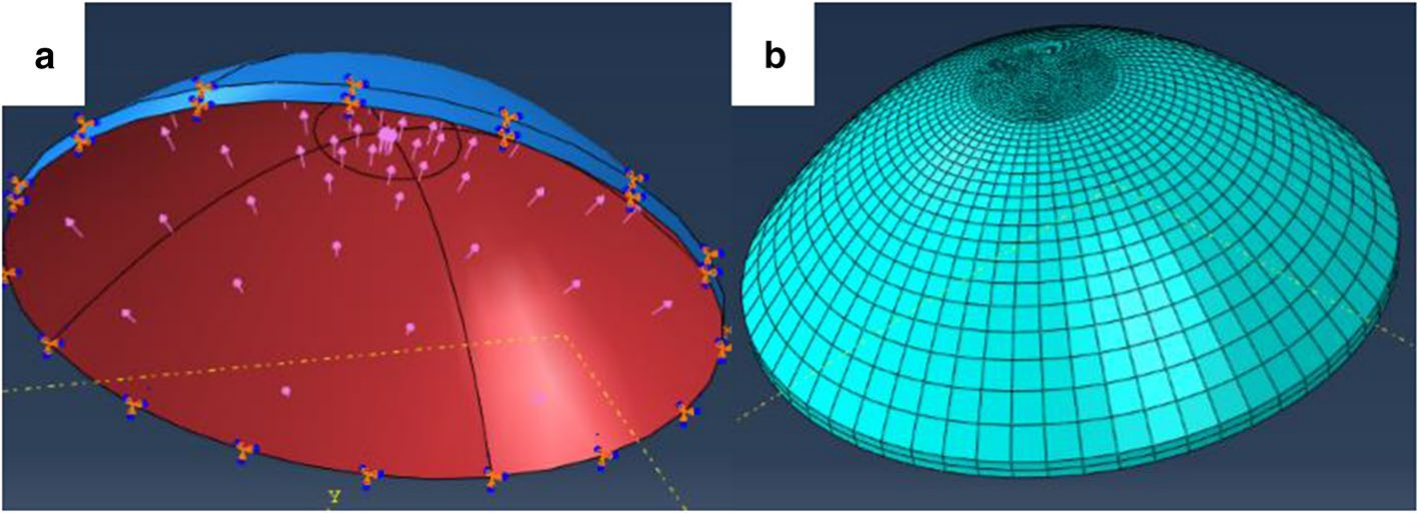
Corneal geometrical model (**a** The geometrical model, load and constraint; **b** corneal meshing results)

To validate the calculated corneal elastic modulus, explicit dynamical finite element method was used to simulate Corvis test. The geometrical model (Fig. 5) was build based on human corneal geometrical pictures of optical coherence tomography (OCT). As corneal topography is measured at a specific intraocular pressure IOP and is distinct from the unloaded shape that would be obtained at an IOP of 0 mm Hg, the undeformed state was solved by a custom finite element model at first. Air puff force was applied on corneal apex as a 30-ms surface traction which was normal distribution and radius, the variation of the amplitude of the air-puff force with time was set according to Ref. [2] which recorded the force with spherical pressure transducer. Cornea was hypothesized to be linear elastic material, the corneal elastic modulus was set to be the calculated modulus and the Poisson’s ratio was set to be 0.49. According to the physical state of the cornea in the Corvis test, a uniform pressure was applied to the inner surface of the cornea according to bIOP measured by Corvis, and the displacements and rotation of limbus are constrained as the displacements and rotation of limbus can be negligible during Corvis measurements. Cornea was meshed with C3D8R mesh and explicit dynamic analysis was used to simulate the measurements. The corneal apical displacements were extracted to compare with experimental data when the displacements were 0.2–0.4 mm. The finite element analysis was conducted on ABAQUS/Explicit 6.12.

### Statistical method

Kolmogorov–Smirnov (K–S) normality test was applied to test the normality of distribution of the rabbits’ and human Corvis parameters in vivo. One-way ANOVA analysis and Spearman correlation test was used to analyze the correlation between Corvis parameters, corneal elastic modulus and IOP. Regression analysis was carried out to establish the relationship between corneal elastic modulus and IOP. Bland–Altman diagram was used to compare the simulated and experimental corneal displacements. All of the statistical analysis was performed using SPSS 21.0 (International Business Machines Corporation, New York, United States of America) and MedCalc 13.0 (Ostend, Belgium), *p *< 0.05 was considered to be statistically significant.

## Results

**Fig. 6:**
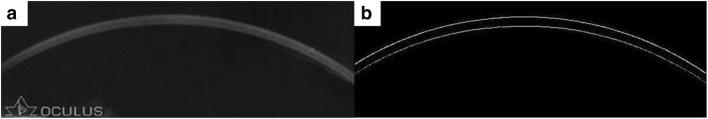
Corneal morphological image (**a**) and result of corneal edge detection (**b**)

Corvis results that “Alignment” and “Pressure Profile” have readings of “OK” were accepted and otherwise repeat measurement was made until the reading was “OK”. For all pictures from Corvis test, corneal edge was detected with threshold segmentation based on canny operator. Figures 6 gives one of the corneal original shape and the results of edge detection. From the result we can find that the method we used to detect corneal edge is effective.

### Results of rabbit corneal Corvis tests in vivo and in vitro

K–S test results showed that all of the rabbits’ corneal Corvis parameters (Table 1) in vivo are normal distribution (*p *> 0.2). In Table 1, SP-A1 is the corneal Stiffness Parameter, DA is the deformation amplitude, A1T and A2T are the time of the first and second applanation respectively, HCT is the highest indentation time, PD and HR is the peak distance and corneal inverse radius when the corneas arrive at corneal highest indentation.

**Table 1:** Result of rabbits’ corneal Corvis test in vivo

	CCT/μm	IOP/mmHg	SP-A1/mN mm^−1^	DA/mm	A1T/ms	A2T/ms	HCT/ms	PD/ms	HR/mm
Mean	384	7.5	16.916	1.17	6.35	21.93	17.48	4.65	5.16
Sd	32	2.1	8.377	0.11	0.14	0.52	0.61	0.19	0.50

**Fig. 7:**
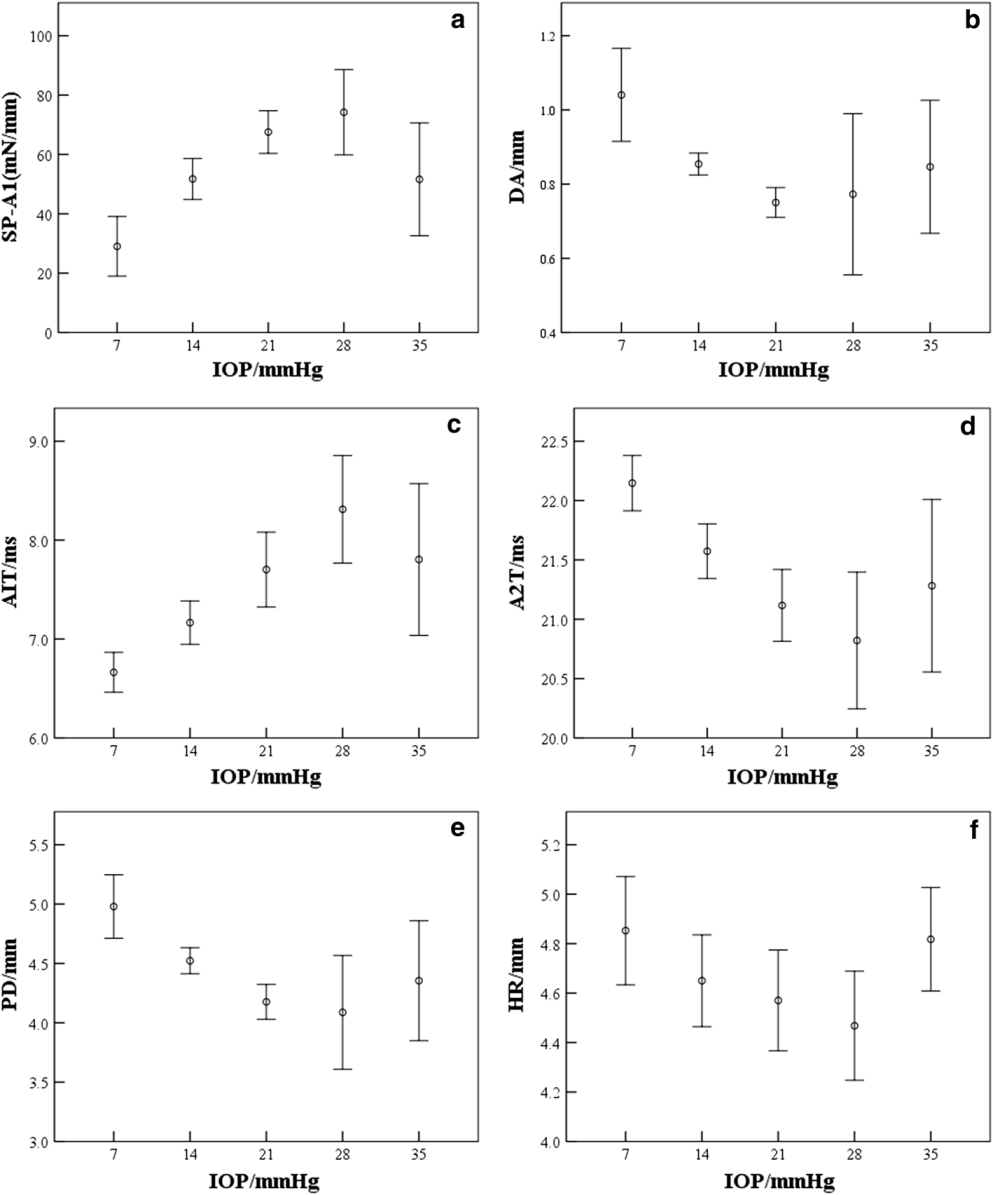
Variations of Corvis parameters with intraocular pressure (**a**–**f** represent the variations of SP-A1, DA, A1T, A2T, PD, HR with IOP, respectively)

Figure 7 gives Corvis parameters that varied significantly with IOP. One-way ANOVA analysis results showed there were significant differences among different IOP groups (p < 0.05) for stiffness parameter (SP-A1), corneal deformation amplitude (DA), the first and second applanation time (A1T, A2T), peak distance (PD) and corneal inverse radius when the cornea arrive at corneal highest indentation (HR). Results of correlation analysis between Corvis parameters and IOP showed that DA, A2T, PD were negatively correlated with IOP (*r *= − 0.497, − 0.443, − 0.475; *p *< 0.05) and A1T, SP-A1 were positively correlated with IOP (*r *= 0.472, 0.444; *p *< 0.05). While the correlation between HR and IOP was not significant (*r *= − 0.108; *p *= 0.375). In addition, we noticed that cornea has obvious vibration under IOP of 35 mmHg and the Corvis parameters showed poorer repeatability and higher standard deviation. Figure 7 shows that Corvis parameters and IOP (7–28 mmHg) were significantly correlated. So corneal elastic moduli at IOP of 7–28 mmHg were calculated in this study.

### Results of rabbit corneal elastic modulus evaluation

**Fig. 8:**
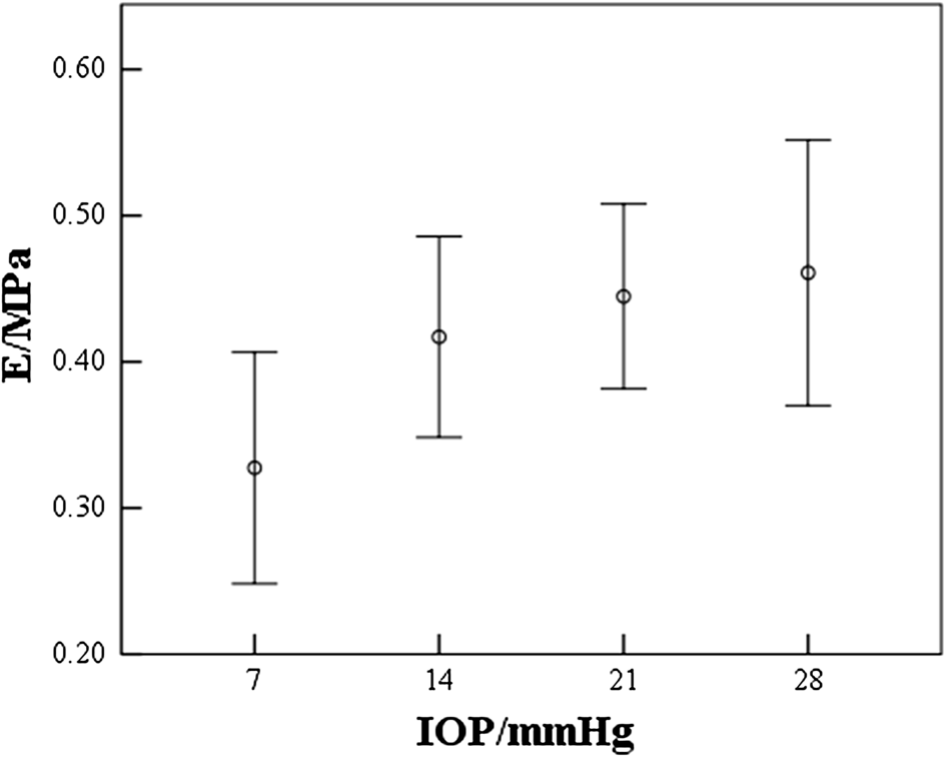
Variations of corneal elastic modulus with IOP

Corneal elastic modulus was calculated according to Eq. 5. The average of the rabbits corneal elastic modulus was 0.24 ± 0.06 MPa (0.16–0.35 MPa). Figure 8 showed that corneal elastic modulus was positive correlated with IOP (*r *= 0.417; *p *= 0.001). The regression equation between corneal elastic modulus and IOP was:

6 $$E = 0.006 \times {\text{IOP}} + 0.306$$

From Fig. 8 we can see that although corneal elastic modulus was positively correlated with IOP overall, the elastic modulus was stable relatively at IOP of 14–28 mmHg, which may show the calculated corneal elastic modulus was less affected by IOP compared to SP-A1 (Fig. 7a).

### Results of the validation of the method by finite element analysis

**Fig. 9:**
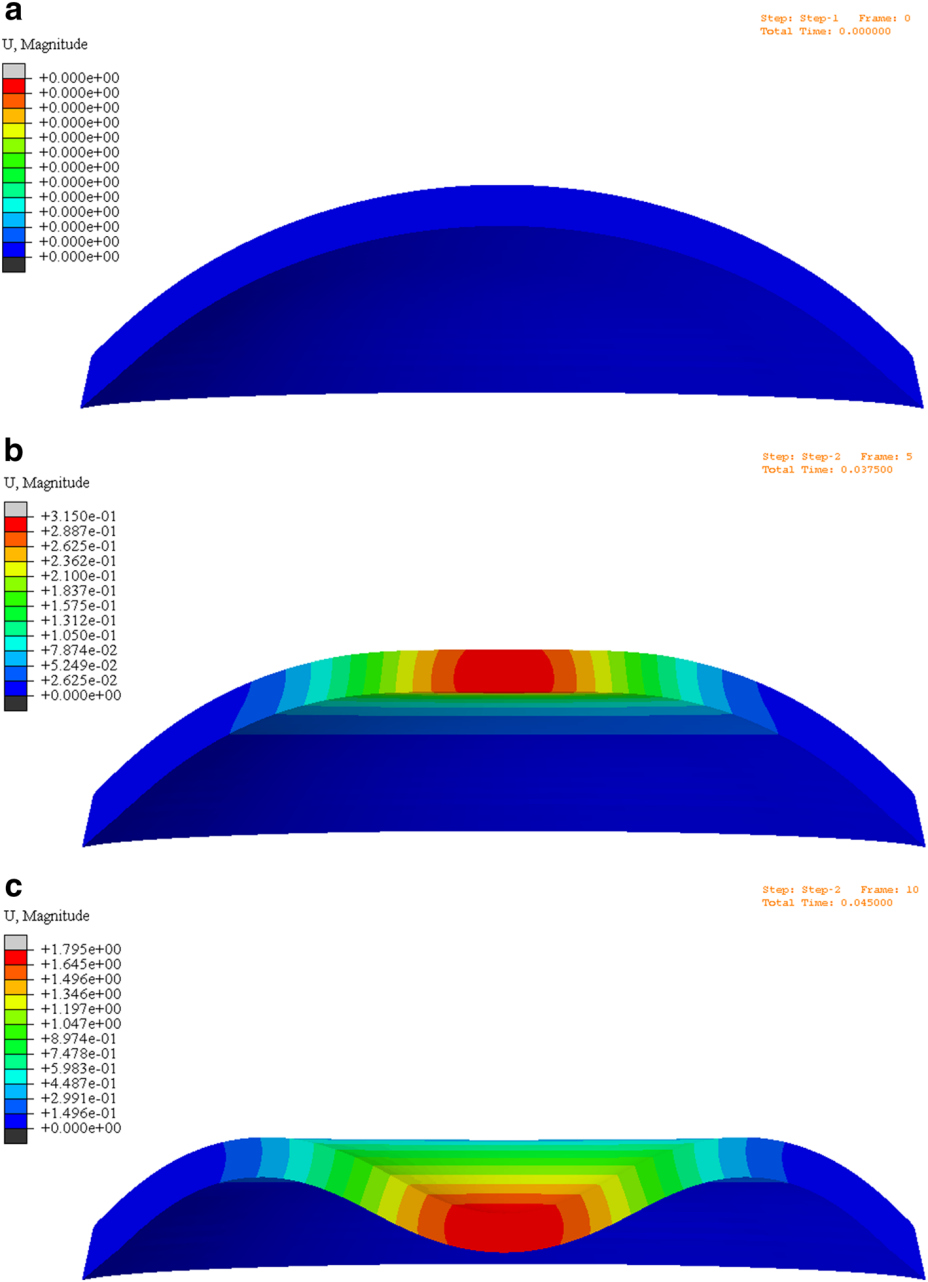
Cornea displacements distribution of the initial (**a**), the first applanation (**b**) and the maximum indentation (**c**) state

Figure 9 exhibits the results of the simulation of Corvis tests using finite element method, and (a)–(c) represent the displacements distribution of the cornea at the initial state, the first applanation state and the maximum indentation state during the Corvis test respectively.

**Fig. 10:**
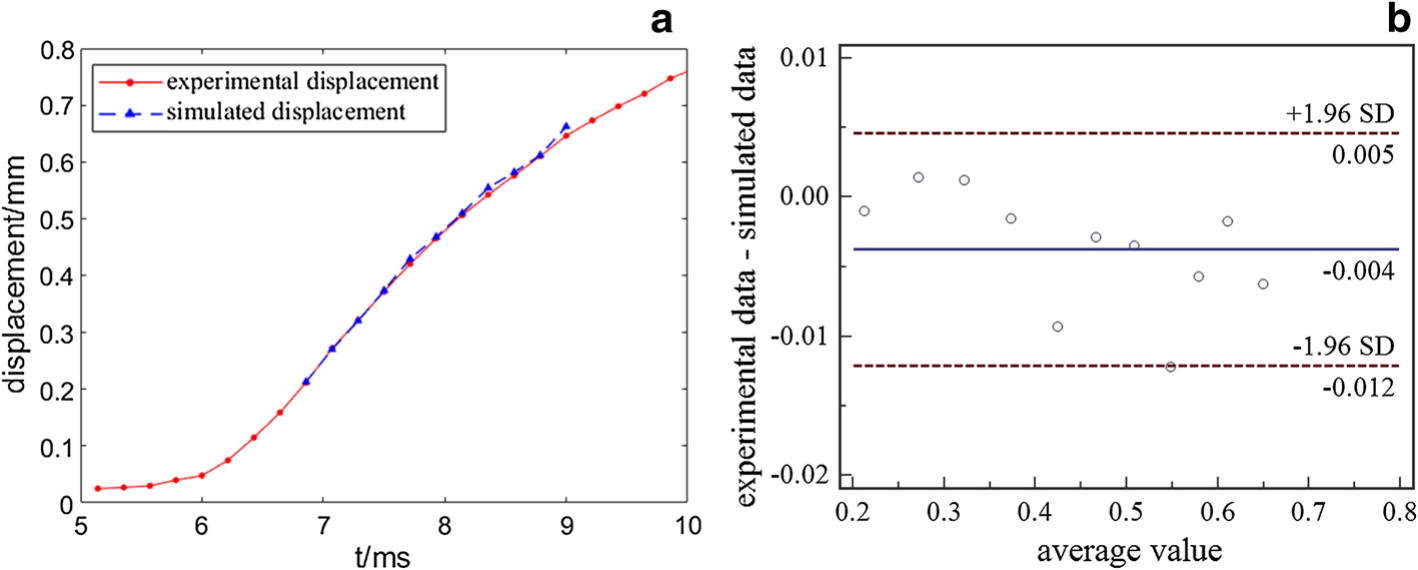
Results of the comparison between simulated and experimental displacements (**a** the experimental and simulated corneal apical displacements; **b** Bland–Altman diagram of the simulated and experimental displacements)

The results of the comparison between the simulated and experimental corneal apical displacements were showed in Fig. 10. Figure 10a is the variations of corneal apical displacements with time and the red and green curve represent the simulated and experimental results respectively. Figure 10b is the Bland–Altman diagram of the simulated and experimental displacements. From the results we can see that the simulation results and the experimental results showed a well coincidence, which indicated that our method can evaluate corneal elastic modulus effectively.

### Results of human corneal Corvis tests and elastic modulus evaluation

The results of human corneal Corvis test were showed in Table 2. All of the parameters were normal distribution, and corneal elastic modulus of healthy young human was 0.22 ± 0.05 MPa (0.14–0.30 MPa).

**Table 2:** Result of human corneal Corvis parameters and elastic modulus

	CCT/μm	IOP/mmHg	SP-A1/mN mm^−1^	DA/mm	A1T/ms	A2T/ms	HCT/ms	PD/ms	HR/mm	E/MPa
Mean	522	14.6	122.528	1.02	7.18	21.05	16.86	4.82	6.96	0.22
Sd	45	2.3	51.277	0.10	0.34	1.40	0.52	0.24	0.96	0.05

Table 3 presents some Corvis parameters that were significantly correlated with corneal elastic modulus, we can see that corneal elastic modulus was positively correlated with A1T and negatively correlated with CCT, DA, HCT, PD and HR. Besides, the correlations between corneal elastic modulus and SP-A1, IOP were also showed in Table 3, we can find the correlation were not significant.

**Table 3:** Correlation between corneal Corvis parameters and elastic modulus

	SP-A1	IOP	CCT	DA	A1T	HCT	PD	HR
*r*	0.061	0.139	− 0.838^a^	− 0.742^a^	0.458^a^	− 0.387^a^	− 0.482^a^	− 0.402^a^
*p*	0.822	0.526	0.000	0.000	0.000	0.000	0.000	0.000

^a^The correlation is significant between parameters

## Discussion

In this study, we provide a method to determine corneal elastic modulus in vivo from the output data of Corvis measurements according to Reissner’s theory [28] which described the stresses and small displacements in shallow spherical shells. The rabbit and human corneas in vivo tests, as well as rabbit eyeballs in vitro tests by Corvis under different IOPs were carried out. The results showed that Corvis parameters such as corneal stiffness parameter, deformation amplitude, applanation time, and peak distance etc. were correlated with IOP. The corneal elastic modulus increased slightly with IOP under physiological IOP in vitro and the correlation between corneal elastic modulus and IOP in vivo was not significant. So, the most important innovation of this study is that a convenient and effective method to determine corneal elastic modulus in vivo based on Corvis test results was established. The method will provide important information for the diagnosis of some ocular diseases and the design of corneal surgery.

The relationship between stress and displacement of shallow spherical shell corneal model has been used in evaluation corneal elastic modulus based on corneal indentation experiments with 3–5 mm diameter cylindrical indenter in recent years [26, 27], and the results showed the possibility of this method to obtain corneal elastic modulus. We noticed that the estimation of tangent modulus in the literatures mentioned above [26, 27] was carried out according to Roark’s formula [29]. In this formula elastic modulus was calculated by interpolation method and the error cannot be ignored when *μ* is not small enough. If we regard Corvis tests as an indentation experiment for the cornea, a method to determine corneal elastic modulus in vivo can be established since corneal deformation and air-puff force can be obtained directly in Corvis tests. As *r*_*p*_ is not small enough, we calculated *μ* and solved *c*_1_ for every test according to Eq. 4 [28] instead of interpolation method. Compared to corneal indentation tests reported by Wang et al. [26], our method based on noncontact Corvis tests which are safer. Compared to the inverse finite element method, this method is more convenient and feasible as we do not need to establish complex corneal geometrical model and conduct abundant calculation. Besides, the simulated displacements using inverse finite element method showed a well coincidence with the experimental displacements, which remind us that the calculated corneal elastic moduli have same effectivity with inverse finite element when taking the error of corneal apical displacements between simulated results and experimental results as objective function. As air-puff force acts on an area around corneal apex, our method is closer to real experiments than Taber’s model in which Corvis test was regarded as a concentrated force acted on corneal apex.

The correlation between corneal Corvis parameters and IOP showed that DA, A2T, PD were negatively correlated with IOP; A1T and SP-A1 were positively correlated with IOP. Similar research was made by Metzler [30] and Bao et al. [31]. The range of Corvis parameters of rabbit cornea are consistent on the whole and the correlation between Corvis parameters and IOP in our results is coincident with their results at IOP of 7–28 mmHg. While the trend of variation is opposite at IOP of 28 mmHg to 35 mmHg. The possible reason maybe that the IOP was monitored at vitreous body in their research while at anterior chamber in our study, and the pressure difference caused the different Corvis parameters values at the same IOP. And the results showed that Corvis parameters present certain instability at anterior chamber pressure of 28 mmHg to 35 mmHg. It reminds us to be attention when evaluate corneal biomechanics of patients with high IOP by Corvis. This hint from Corvis measurements of eyeballs should be further verified from clinical data.

To the authors’ knowledge, there was few studies have reported Corvis parameters of rabbit cornea in vivo. The coefficient of variation (sd/mean) of Corvis parameters showed a good consistency with the results reported by researches about the Corvis parameters of normal human cornea, which indicated that the Corvis parameters of rabbit cornea has a similar repeatability with human Corvis parameters. From Eq. 6 and Fig. 8, rabbit corneal elastic modulus ranged from 0.16 to 0.60 MPa when IOP increased from 7 to 35 mmHg. Corneal elastic moduli were usually obtained from corneal uniaxial tensile test or corneal inflation tests, and the range of normal rabbit corneal elastic modulus ranged from 0.1 to 0.36 MPa in different studies [7, 24, 32–35]. Corneal elastic modulus extracted from this study has the same magnitude with the results reported by the literature. Besides, corneal elastic modulus was found positively correlated with IOP, and corneal inflation tests of human cornea showed a similar positive correlation between corneal elastic modulus and IOP [36]. These results remind us cornea present nonlinear elastic properties under IOP of 7–35 mmHg. It has been known that Except IOP, corneal thickness and corneal curvature radius maybe also the main factors when determine corneal elastic modulus. From Eq. (3) and Eq. (5), we have considered the influence of corneal thickness and curvature radius in the computation of the elastic modulus. To analyze whether our calculated elastic modulus has screen out the influence of corneal thickness and curvature radius, we calculated the correlation between elastic modulus and thickness, curvature radius. Results showed the correlation between corneal elastic modulus and corneal thickness (*r *= 0.041, *p *= 0.823), corneal curvature radius (*r *= 0.115, *p *= 0.531) in vivo were not significant, in vitro results also showed our calculated elastic modulus was less affected by corneal thickness (*r *= 0.476, *p *= 0.086) and curvature radius (*r *= 0.253, *p *= 0.384). So, we considered that the method proposed in this study to extract corneal elastic modulus based on results of Corvis tests is effective for rabbit cornea.

As human cornea is too precious to get its biomechanical properties, human corneal elastic modulus was usually obtained from biomechanical tests in vitro of cadaver eyes or transplant cornea at present. Wollensak et al. [37] measured human corneal elastic modulus by corneal uniaxial tensile tests and got that the elastic modulus of human cornea was 0.8 MPa. Elsheikh et al. [36, 38] measured corneal biomechanical properties by corneal inflation experiments and got the normal range of human corneal elastic modulus was 0.16–0.8 MPa. Human corneal elastic modulus extracted from this study was 0.14–0.30 MPa, which was coincident with above experiments results at the magnitude. Besides, with the development of corneal biomechanical tests such as Corvis test and corneal indentation test, corneal biomechanical properties can be obtained by inverse finite element methods or other methods based on these results conveniently, and the evaluated corneal elastic modus ranged from 0.05 to 0.4 MPa [2, 24, 34, 39], which also showed a well coincidence with our results. Therefore, the method proposed in this study to evaluate corneal elastic modulus was feasible and effective.

In the latest version of Corvis software, corneal stiffness parameter (SP-A1) has been provided. Besides, Wang [2] has also extracted a parameter termed *S*_TSC_ to reflect corneal elastic properties. Both of the two parameters reflect corneal stiffness. From Fig. 7a, SP-A1 increased significant with IOP while corneal elastic modulus was stable relatively (Fig. 8) at IOP of 14–28 mmHg. From Table 3 we can see the correlation between SP-A1and corneal elastic modulus was not significant. These results indicated that SP-A1 was more influenced by IOP than corneal elastic modulus, and it is not enough to evaluate corneal elastic property according to SP-A1. In this study, *S*_TSC_ was also calculated and results showed *S*_TSC_ increased significantly with IOP. The calculated corneal elastic modulus from Corvis measurements in vivo also showed less significant correlation with IOP (*r *= 0.139, *p *= 0.526) than SP-A1 and *S*_TSC_ (*r *= 0.429, 0.437; *p *< *0.05*), which indicated that corneal elastic modulus calculated in this study may be less affected by IOP when the IOP is in physiological range than SP-A1 and *S*_TSC_. Even the principle of the stronger correlation between S_TSC_, SP-A1 and IOP, and the weaker correlation between corneal elastic modulus and IOP in our study need to undertake further analysis, our calculated results showed that our method is more reliable.

Corneal elastic modulus has been reported to be lower in keratoconus patients [2] and shown increased after corneal refractive surgery [7]. Corneal biomechanical properties also changed significantly after corneal transplantation [4–6]. One could calculate corneal elastic modulus, for example, based on our method from Corvis results thoroughly. Calculated corneal elastic modulus may provide important information for the diagnosis of keratoconus, the prevention of corneal ectasia after corneal refractive surgery. In addition, from Figs. 7 and 8, corneal elastic modulus showed similar trend with A1T and SP-A1, and opposite trend with DA, A2T, PD, when IOP increased from 7 to 35 mmHg. The significant correlation between Corvis parameters and corneal elastic modulus (Table 3) showed similar results for Corvis measurements in vivo. These indicated that one can estimate whether corneal elastic modulus is in normal range according to Corvis parameters roughly. This study makes it possible to diagnose abnormal corneas more timely and effectively based on the combination of corneal morphological examination results and corneal elastic modulus.

The limitation of this study is that cornea was regarded as a spherical shell and the ununiform corneal thickness and curvature were ignored which may have influence on the evaluation of corneal elastic modulus, while the simulated corneal apical displacements based on the calculated elastic modulus showed a well coincidence with the experimental results, which indicated that our simplification was acceptable; In addition to corneal elastic modulus, corneal viscoelastic and nonlinear elastic properties are also important aspects of corneal biomechanics, so study should be made on the method to determine corneal nonlinear elastic and viscoelastic properties based on Corvis tests in the future, in which the concrete results of finite element calculation, such as deformation amplitude, flattening time and peak distance will be used to match with the experimental data.

## Conclusions

From this study we can conclude that the method we proposed to determine corneal elastic modulus based on Reisner’s theory is convenient and effective, and the calculated corneal elastic modulus was less influenced by IOP. The clinical significance in the prognosis of eye surgery or in patients with keratoconus remains to be proved by abundant clinical data. In the future, we may calculate corneal elastic modulus of normal and keratoconus patients according to this method to explore its value in diagnosis of early keratoconus.
